# The effect of the Covid-19 pandemic on patient visits to the emergency department and hospitalizations in medical wards in an Israeli medical center

**DOI:** 10.1186/s13584-021-00495-5

**Published:** 2021-11-01

**Authors:** Yael Frenkel Nir, Yuval Levy, Amit Gutkind, Ehud Grossman

**Affiliations:** 1 Medical Management Department, The Chaim Sheba Medical Center, Tel Hashomer, Israel; 2 Internal Medicine Wing, Sheba Medical Center, Tel Hashomer, Israel Affiliated To Sackler Faculty of Medicine, Tel-Aviv University, Tel Aviv, Israel; 3 Israel Defense Forces, Israel Medical Corps, Ramat Gan, Israel

**Keywords:** Covid-19, Medical wards, Emergency Department, Hospital admissions

## Abstract

**Background:**

The Covid-19 pandemic began in Israel on February 2020. Between February and October 2020, 2 periods of lockdown were imposed on Israeli population.

**Objective:**

To assess the effect of the Covid-19 pandemic on visits to the emergency department (ED) and on hospitalizations in medical wards in Israel’s Chaim Sheba Medical Center, and to compare the effect during the first and second lockdowns.

**Methods:**

Data regarding the number of visits of non-Covid-19 patients to the ED and the number of admissions to the medical wards, were extracted from the computerized system of the hospital. Data were analyzed for patients' characteristics, length of stay in the medical wards, in hospital mortality and the rate of 7 and 30 days re-hospitalization, and compared to the same period during 2019.

**Results:**

Total visits to the Sheba ED during March-October decreased by 18.5%. The most dramatic decrease occurred during the first lockdown. The number of patients admitted to the Sheba medical wards decreased by 28% (*P* < 0.05). The length of stay decreased from 3.69 days during 2019 to 3.42 days during 2020 (*P* < 0.01). The most pronounced decrease in the length of stay was observed during the second month of the first lockdown. During the pandemic, hospitalized patients at Sheba were older and were less likely to be males. The in-hospital absolute non-COVID mortality decreased from 913 to 858 respectively.

**Conclusions:**

The Covid-19 crisis emphasizes the role of medical wards in the care of complex patients. Medical wards in Israel were at the frontline of Israel's battle against this pandemic, while continuing to treat very complex non-Covid patients. To avoid burnout of the medical staff who treat very intensively complex patients, we believe these wards should be strengthen with specialists having expertise in treating these patients. Due to our insights, the Sheba medical Center is now redesigning the concept of how intensive care beds should be managed in a big tertiary center.

## Introduction

The corona virus disease 2019 (Covid-19) Pandemic began in Israel on February 20 this year. Sheba Medical Center (SMC), the biggest hospital in Israel, was the first to hospitalize Covid-19 patients. During the months March to October 2020, 1,268 Covid-19 patients were treated in the SMC for a total of 9726 hospitalization days. The hospital was quickly prepared to receive hundreds of patients with more than half of the medical wards in the hospital converted to Covid-19 wards.

The outbreak in Israel surged on March leading to some restrictions and later to a full-scale lockdown from the middle of March to the end of April [1]. After relieving many restrictions and reopening the education system, the second surge began in mid-June growing to its peak in mid-September. This led to the second lockdown during September- October 2020.

In Israel, there was a significant decline in the visits to the emergency department (ED) during the first wave of the Covid-19 outbreak [2]. Hospital occupancies decreased and the number of elective procedures including cardiac surgeries declined [1–3].

Mendlovic et al. showed a decrease in the number of patients visiting the ED and in the number of admissions to medical wards during the outbreak. The characteristics of the admitted patients were similar, but the hospital length of stay was shorter. They concluded that an infectious disease outbreak has an effect on uninfected admitted patients [4].

The effect of the Covid-19 on non-Covid patients incommoded health care institutes all around the world [5–10]. Decreased activity has been observed not only in elective procedures but also in the acute medical care. In France, a marked decrease in hospital admissions for acute myocardial infarction was observed following the lockdown, irrespective of patient characteristics and regional prevalence of Covid-19 [11]. In Norway a significant reduction in the number of admissions for stroke and transient ischemic attacks was observed during the lockdown due to the Covid-19 pandemic [12].

Sheba Medical Center used a successful triage method separating Covid-19 positive from negative patients and maintained the regular hospital clean of corona virus, allowing for safe treatment continuation of non-Covid-19 patients [13].

The aim of this study was to assess the effect of the Covid-19 pandemic on the visits to the ED and on the hospitalizations in medical wards, and to compare the effect during the first and second lockdowns. Our experience may help to better prepare for the next Covid-19 wave in Israel and the up-coming winter.

## Methods

Data regarding the number of visits of non-Covid-19 patients to the Sheba medical center's ED and the number of admissions to the hospital's medical wards, were extracted from the computerized system of the hospital. Data were analyzed for patients' characteristics, length of stay in the medical wards, in hospital mortality and the rate of 7 and 30 days re-hospitalization. We also checked for the number of diagnoses recorded for each patient on discharge as a rough estimation for patients' severity or complexity.

Data were extracted for the whole period (March to the end of October 2020) and were compared to the same period during 2019. To compare the first and second lockdown, data were extracted for each month separately.

### Statistical analysis

Descriptive statistics were performed to assess characteristics of the study population. Comparison of quantitative variables in two independent groups were performed using the t test. Two-sided *P* values of < 0.05 were considered significant.

## Results

### Visits to Emergency departments and the rate of hospitalization to medical wards

**Fig. 1:**
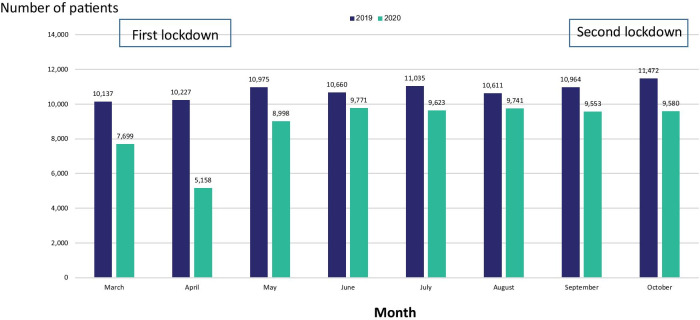
Comparison of visits to Emergency Department by month, during the years 2019–2020

Total visits to the ED during the months March-October decreased from 86,081 in the year 2019 to 70,123 in 2020 (18.5% reduction). The most dramatic reduction occurred on April during the first lockdown (Fig. 1). The hospitalization rate from the ED to all hospital wards decreased significantly from 35.41% in 2019 to 31.84% in 2020 (*P* < 0.01).

Compared to March-October 2019, the number of patients admitted to medical wards decrease during March-October 2020 by 28% from 19,012 to 13,691(Table 1, *P* < 0.05). The number of admissions to medical wards decreased by 41% during the first lockdown and only by 31% during the second lockdown (Table 1).

**Table 1:** Number of admissions to the medical wards

Period	Year		Change (%)
	2019	2020	
March–October	19,012	13,691	− 5,321 (− 28%)
March–April (1st lockdown)	4,800	2,815	− 1,985 (− 41%)
May–August	9,475	7,606	− 1,869 (− 20%)
September–October(2nd lockdown)	4,737	3,270	− 1,467 (− 31%)

**Fig. 2:**
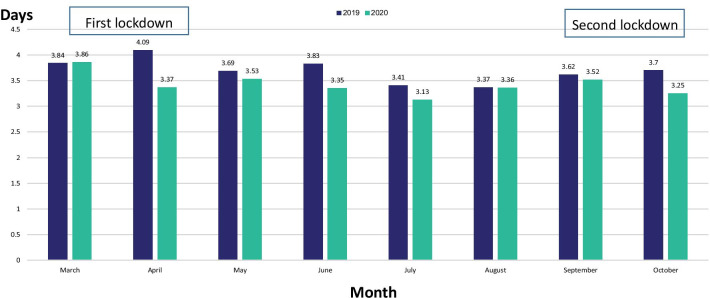
Comparison of length of stay in the medical wards by month, during the years 2019–2020

The length of stay in the medical wards decreased from 3.69 days during March -October 2019 to 3.42 days during March -October 2020 (Table 2; *P* < 0.01). The most pronounced decrease in the length of stay was observed during the second month of the first lockdown (Fig. 2).

**Table 2:** patients' characteristics and data on hospitalizations

Parameter	Year	
	2019	2020
Age (years)	69.8	70.4
Males (%)	54.8	51.2
Length of stay (days)	3.69	3.42
Number of diagnoses	5.66	7.66
In hospital mortality (n)	913	858
Rate of in hospital mortality (%)	4.75	6.38
Rate of 7-days re-admission (%)	7.08	5.44
Rate of 30-days re-admission (%)	15.59	12.16

### Patients' characteristics

During the pandemic, hospitalized patients in the medical wards of the SMC, were older than the year before (70.4 years vs. 69.8, *P* = 0.019), and were less likely to be males (Table 2). The average number of diagnoses for patient on discharge has increased from 5.66 to 7.66 (*P* < 0.01). The non-COVID mortality rate in the medical wards increased from 4.75% during the months March-October 2019 to 6.38% during the same months in 2020. However, the in hospital absolute mortality decreased from 913 to 858 deaths respectively (*P* < 0.05).

## Discussion

In the present study, we showed that during the Covid-19 Pandemic in Israel, fewer patients came to the Sheba medical center (SMC) ED and the percent of admission to the medical wards decreased. Thus, the number of patients hospitalized in our non-Covid medical departments decreased. It is important to state that we had fewer medical wards available for non-Covid patients, since some of them were transformed into Covid wards. In addition, one must stress that during at least 3 months of the study period, Israel was under strict lockdown restrictions, meaning that people came to the hospital mainly for emergency conditions.

The main effect on the visits to the ED and the number of hospitalizations was observed during the first lockdown and was less pronounced during the second lockdown. It seems as if patients preferred to stay at home during the first lockdown because of fear of Covid-19 contamination. As time passed, people got used to the new situation (living with the pandemic) and started returning to the ED for less severe conditions. We used a triage system in our ED that helped us separate patients with suspected Covid infection from non-Covid patients [13], but it seems it took time until this fact became known to the public and people began to trust, that the non-Covid departments, are actually safe and clean from corona.

Unlike the findings of Mendlovic J. et al. [4], we showed that those who were hospitalized were older and had more diseases (number of diagnoses per patient on discharge). This indicates that during the pandemic, only patients with complex diseases that needed urgent treatment were hospitalized. This also led to an increased mortality rate in the medical wards during this period. However, the absolute number of deaths decreased compared to the same period in the previous year. It is noteworthy that the decreased in hospital mortality was related to non-Covid-19 patients hospitalized in the medical wards. However, when adding the absolute mortality found in the medical wards converted to Covid-19 wards, the absolute mortality of the whole medical wing, increased during 2020 by about 10% as compared to 2019. Data from the general population in Israel showed that during March-September 2020, the gross mortality rate was 3% higher than in the previous year and 4% higher than the average for the years 2017–2019 [14]. This increase in mortality may be explained by the excess Covid-19 mortality. At the same time, the percentage of in-hospital deaths was slightly lower than during previous years. Combining our data and the data from the general population may suggest that during the pandemic, many patients that were non-Covid-19 patients and were reluctant to come to the hospital for acute care, were treated in the community and therefore it is resendable to speculate that there was also a shift of deaths from the hospitals to the community. This could be established only after the Israeli Ministry of Health (MOH), will publish the list of community causes of deaths for 2020.

Although hospitalized patients had more severe morbidity as reflected by older age and higher number of diagnoses per patient, the length of stay in these wards was shorter than in the previous year. Similar observations were described by others [4]. The decrease in length of stay could be related to the better availability of community facilities. During the pandemic, the Israeli MOH increased the number of beds in some of the residential long-term care facilities, making it easier for hospitals in Israel to discharge patients earlier. It should be noted that the number of long-term care beds in Israel, has decreased per capita in the last decade, a fact that makes discharge from medical wards difficult on every day basis. In addition, the increased demand on the limited number of non Covid-19 medical beds, led the medical staff to work even harder and try to discharge patients earlier. The higher availability of beds in long-term care facilities, made this effort possible.

The shorter hospitalization time in the medical wards did not lead to an increase in readmissions, as one might suspect. Just the opposite. We found that the rate readmissions to the medical wards (both 7 days and 30 days after discharge), has actually decreased during the study period. It is not clear whether this was a result of the general populations' reluctance to return to the hospital during the pandemic, or to a better care given to these patients in the community setting. Our results may suggest that during the pandemic, the very hard work of the hospital medical staff and the better availability of community services, enabled us to deliver high quality medical care even with less available non-Covid-19 beds. It is noteworthy that despite the shortage of medical beds, the absolute number of in-hospital deaths and the rate of re-admissions decreased.

This study has some limitations. First, we do not have information on the community services and the rate of out of hospital mortality in our area. Second, we used indirect assessment of the morbidity of the hospitalized patients (i.e. number of diagnoses on discharge). Third, we do not know the exact impact of the intense work of the medical personal on their emotional state and overall burnout. We are now beginning a study to evaluate the impact of the hard and stressful work during the Covid-19 pandemic on the staffs' burnout.

To sum up our findings: The Covid-19 pandemic reduced the number of referrals to the ED, as well as the rate of hospitalization. Patients hospitalized were more complex and the percentage of deaths increased although the absolute number of in-hospital deaths decreased. Even though the length of hospitalization was shorter, readmission rate decreased. As in many other outbreaks in the past, the medical wards are always in the front-line of theses battles. We believe that intensification of the medical wing with more specialists (especially those with intensive care capabilities), will enable the treatment of complex patients with better outcomes. We still need to evaluate the impact of increased burden on the burnout of the medical staff in these wards, which we believe was quite high during this year. Hospital managements and leaders in health policy will need to think on how these wards should look like in the next decade in order to make them and their staff, better prepared for future challenges.

## Conclusions

Medical wards in Israel were at the frontline of Israel's battle against this pandemic, while continuing to treat very complex non-Covid patients. To avoid burnout of the medical staff who treat very intensively complex patients, we believe these wards should be strengthened with specialists having expertise in treating these patients.

## Data Availability

The datasets during and/or analyzed during the current study available from the corresponding author on reasonable request**.**

## Abbreviations

ED: Emergency Department
SMC: Sheba Medical Center
MOH: Ministry of Health
